# The use of complementary and alternative medicine (CAM) in Europe

**DOI:** 10.1186/s12906-020-02903-w

**Published:** 2020-04-06

**Authors:** Erlend L. Fjær, Erling R. Landet, Courtney L. McNamara, Terje A. Eikemo

**Affiliations:** Centre for Global Health Inequalities Research (CHAIN), Department of Sociology and Political Science, Norwegian University of Science and Technology (NTNU), Trondheim, Norway

**Keywords:** CAM use, Socio-economic position, health care systems, Europe

## Abstract

**Background:**

While the use of complementary and alternative medicine (CAM) has become increasingly popular in western societies, we do not understand why CAM use is more frequent in some countries than in others. The aim of this article is to examine the determinants of CAM use at the individual and country-level.

**Methods:**

Logistic multilevel regressions were applied analyzing data from 33,371 respondents in 21 European countries (including Israel) from the seventh round of the European Social Survey. We examined CAM in terms of overall use and also dichotomized treatments into physical and consumable subgroups.

**Results:**

At the individual level, we found CAM use to be associated with a range of socioeconomic, demographic and health indicators. At the country level, we found that countries’ health expenditures were positively related to the prevalence of overall and physical CAM treatments.

**Conclusions:**

A common predictor for CAM use, both at the individual (in terms of education and financial strain) and country-level (in terms of health expenditures per capita), is greater resources.

## Background

In contrast to mainstream or conventional medicine, which typically has its roots in modern science (i.e. biomedicine), complementary and alternative medicine (CAM) encompasses a variety of alternative treatments that have historic origins outside of, and are used in combination with, conventional medicine [1, 2].

The use of CAM treatments, such as acupuncture, homeopathy, and chiropractics, has become increasingly popular in western societies [3–5]. For example, in the US, the use of CAM increased rapidly during the 1990s. The estimated number of visits to CAM practitioners in 1997 exceeded the projected number of visits to all primary care physicians in the US by an estimated 243 million [6]. In Europe, France and Germany were found to have the highest prevalences of CAM use of 8 European countries in 1992, with 49 and 46% respectively of the populations having used some form of CAM [7].

Previous single-country studies have shown that there are differences in the demographic characteristics and health status of users of CAM and non-users [5]. For example, females, those in higher socioeconomic groups and those of middle age, have all been found to be more frequent users of CAM [8, 9]. More recent work has examined the health-related and sociodemographic determinants of CAM treatments specifically in Europe, finding that use of CAM is greater among those with health problems, and more common among women and those with a higher education [10].

Studies thus far, however, have not comprehensively examined why CAM use is more prevalent in some countries when compared to others. This article is therefore the first to do so, using a pan-European data set from the seventh round (2014) of the European Social Survey [11].

In this study, we examine CAM use across 21 European countries (including Israel) in reference to a diverse set of individual and country-level determinants. Specifically, we examine the determinants of CAM use among individuals according to socioeconomic and demographic characteristics, health, health care use and perception of the healthcare system. There is, to our knowledge, no research on what macro-factors might explain the differences in CAM use between countries [3, 4]. We draw on health care systems literature [12] to provide a basis for considering why some indicators should be examined more closely.

Two such macro-factors are GDP per capita and health expenditure. The idea is that richer countries and countries with higher health expenditure are more likely to have integrated CAM treatments into their health care system. Poland, Hungary, Lithuania, Estonia and the Czech Republic for example, all rank at the lower end of GDP per capita [13]. These countries have relatively few CAM treatments reimbursed through health insurance [14]. In Poland, acupuncture is reimbursed, but only for treating chronic pain. In Hungary, some procedures are reimbursed, but the bulk of payments must be made out-of-pocket. In wealthier countries with higher health expenditures, like Switzerland, Norway, Sweden, Denmark and the Netherlands, a greater number of CAM treatments are reimbursed, and integrated into the established health care system [14]. For example, a survey in 2008 found that around 50% of Norwegian hospitals provided some form of CAM, mostly acupuncture [15]. A survey from 2007 found that a third of the people who reported to have used CAM, had the treatment done by a traditional health care practitioner [16]. In short, wealthier countries with higher health care expenditures seem to have integrated CAM treatments into their health care systems to a greater degree than the countries with lower GDP per capita. Whether this integration makes CAM treatments more accessed by the public, remains to be tested.

Other factors that might influence the prevalence of CAM use at the country-level is the density of doctors, gatekeeping functions of the health care system and the price of out-of-pocket payments in primary health care. These factors have been found to be important in work evaluating the accessibility of health care in Europe [12, 17–19]. The idea is that since these conditions have previously been linked to healthcare accessibility issues, they may also provide an incentive for individuals to seek CAM treatments.

There is currently no established way of categorizing or analyzing CAM treatments in social research. Some studies combine a variety of CAM treatments into one overall variable [5], while others choose to categorize treatments into analytical subgroups [10, 20–22]. Davis and colleagues [21] for example, categorize CAM use into *practitioner-based* and *self-administered* treatments. Examples of practitioner based treatments are chiropractics and acupuncture whereas self-administered treatments include products such as natural supplements (vitamins, herbals and minerals), in addition to self-practice activities like yoga and meditation. Other studies [20, 22], by contrast, characterize treatments on the basis of domains described by the National Center for Complementary and Alternative Medicine (NCCAM). These include (1) *whole medical systems* (e.g. acupuncture), (2) *mind-body medicine* (i.e. various spiritual, meditative, and relaxation techniques), (3) *biologically-based systems* (e.g. vitamins and nat ural products), (4) *manipulative and body-based practices* (e.g. massage, chiropractics, and osteopathy), and (5) *energy medicine* (e.g. Reiki therapy) [22]. In this study, we utilize a comprehensive indicator of CAM which combines a variety of types of CAM use into one variable. However, we also make a distinction between physical and consumable treatments, where the former involves the physical manipulation of the body (and includes treatments such as chiropractics) and the latter involves the consumption of a treatment (and includes treatments such as homeopathy). This categorization provides a useful way of comparing whether there are differences in the importance of determinants according to type of CAM use. This distinction also aligns somewhat with Davis and colleagues’ paradigm of practitioner-based versus self-administered treatments. This is because all our physical treatments are also practitioner-based, although our consumable treatments are, according to Davis et al. [21], self-administered.

## Methods

This study was based on data from the seventh round of the European Social Survey (ESS) (European Social Survey, 2014), which includes data from 40,185 respondents in 21 countries: Austria, Belgium, Czech Republic, Denmark, Estonia, Finland, France, Germany, Hungary, Ireland, Israel, Lithuania, Netherlands, Norway, Poland, Portugal, Slovenia, Spain, Sweden, Switzerland and United Kingdom. The data was collected in face-to-face interviews with individuals aged 15 and above living in private households. In this study we included respondents aged 25 and above who were not in education, with non-missing values on included variables. By doing so, we only included respondents that are likely to have completed their education, as those below 25 have often not yet completed their education [23]. Capping at age 25 and removing respondents in education would also remove systematic biases due to differences in the countries’ educational systems and practices. That left 33,371 respondents. The response rates were similar to previous rounds of the ESS, and ranged from 31% in Germany to 68% in the Czech Republic [11].

### Dependent variables

**Fig. 1:**
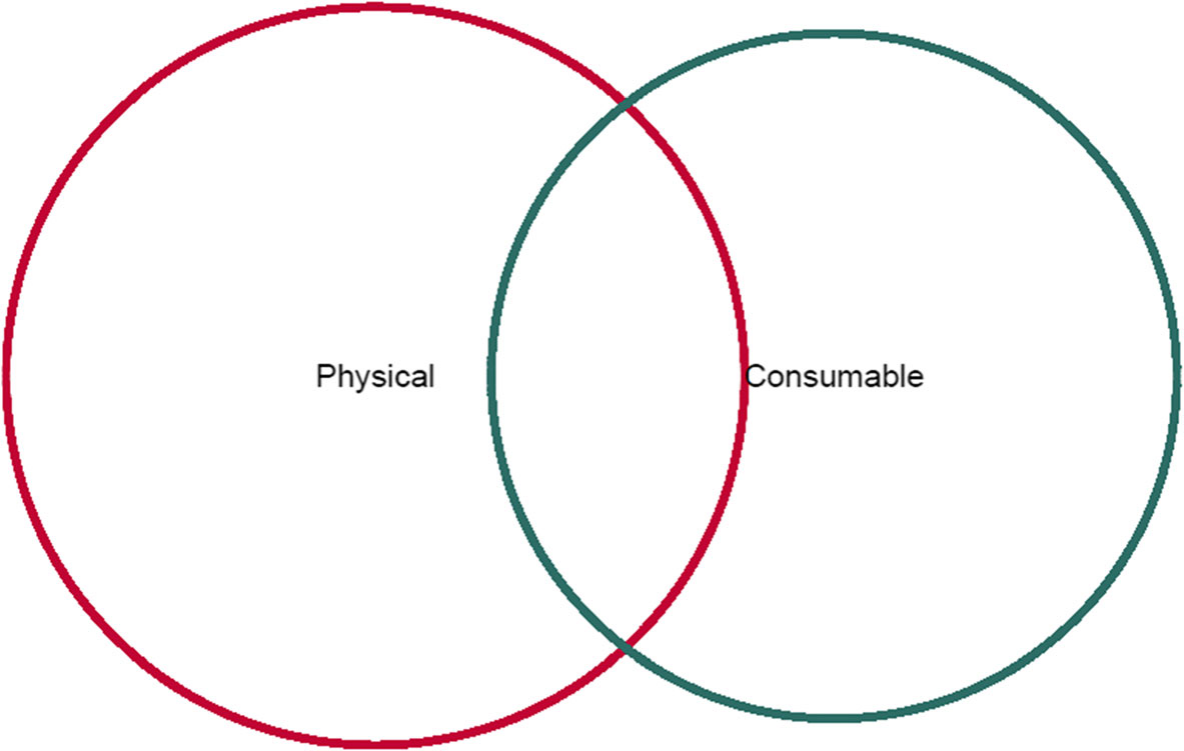
Venn diagram of the three categories of CAM

Respondents were asked if they had used any of 12 different treatments for their own health in the past 12 months. These were acupuncture, acupressure, chiropractics, osteopathy, homeopathy, herbal treatment, reflexology, Chinese medicine, hypnotherapy, massage therapy, physiotherapy, and spiritual healing. Of these, we included the seven first listed treatments in our analysis. The responses were grouped into two categories of CAM use, and one overall measure, combining the two. We use the analytical group of physical to refer to treatments that involve physically manipulating the client’s outer body. It includes the practices acupuncture, acupressure, chiropractics, osteopathy, and reflexology. The consumable group on the other hand, involves the partaker to ingest something that has the purpose of promoting health or well-being. This group contains the practices homeopathy and herbal treatment. We did not include massage therapy and physiotherapy from the physical group due to high prevalence rates and physiotherapy as it historically has been recognized as a part of biomedicine [24, 25], making it a conventional treatment, and not CAM. As the respondents could mark having used more than one treatment, there were only slight overlap. Around 11% of the sample had used at least one physical treatment, about 9.5% had used any consumable treatment, and 2.54% had used both. This is illustrated in the Venn diagram in Fig. 1. However, we still found significant differences in the results between the two subgroups, and therefore we kept them unchanged.

### Socioeconomic and –demographic explanatory variables

- *Gender* was dummy coded, with females assigned the value one, and men as reference.
- *Age* was coded as three 20 year age groups (25–44, 45–64, and 65+).
- *Education* was classified in three categories according to the International Standard Classification of Education (ISCED). The lower educated category included respondents with less than upper secondary education and were used as reference. The middle group with upper secondary education were grouped and distinguished from the higher educated with tertiary education.
- Income groups were identified by the concept *financial strain*; how they felt about their household income. We grouped respondents in three categories; those finding it difficult or very difficult to manage on present income, those coping on present income, and those living comfortably on present income who were used as reference.
- The respondents’ *main activity* was measured by grouping the unemployed or houseworking, and using them as reference, compared to people in paid work, the retired, and the permanently sick or disabled. We dropped the respondents who were in education, in community or military service, without ‘or’ reported ‘other’.

### Health, and use of health care explanatory variables

- *GP/ medical specialist use* measures which of the health professionals the respondents had discussed their health with. Respondents who had not seen a doctor were grouped, and used as reference (0). Respondents who had seen a General practitioner, but not a specialist were grouped as 1. Finally, respondents who had seen a specialist (excluding dentists) were grouped as 2.
- *Unmet need* measures whether respondents were unable to get a medical consultation or the treatment they needed in the past year, while those who did not report unmet need were used as reference.
- Respondents who ranked their satisfaction 4 or lower on a scale from 0 to 10, were treated as being *dissatisfied with overall state of health services* in their country (1). Those responding with the values 5 or higher were used as reference (0).
- *Self-reported health (SRH)* was dichotomized, where the responses ‘very good’, and ‘good’ were coded as good health and used as reference, while ‘fair’, ‘bad’, or ‘very bad’ health were coded as poor health.
- Respondents who reported being hampered in any way by any longstanding illness or disability, infirmity or a mental health problem were treated as having a *longstanding health problem*, while those who did not were used as reference.

### Multilevel analysis

In this study, we employed four country-level variables. Health expenditures per capita, out-of-pocket payments, and physician density were collected from The World Bank [13, 26, 27]. The data was primarily from 2014, but supplied with prior years where missing values were found. A variable measuring gatekeeping in primary health care was constructed based on data from the the Organisation for Economic Co-operation and Development (OECD) [28] supplemented by a policy brief for the European Commission [29], for the missing values of OECD’s report. Countries that were in a gray zone were regarded as having a gatekeeping function in primary health care with the value 1, those without gatekeeping were used as a reference with the value 0. Examples of countries in a gray zone are countries with incentives for gatekeeping, like France, Germany, Switzerland, and Poland. All values of country-level variables for each country is presented in the Additional file 1.

For the analysis, we applied logistic multilevel modeling, and used country as the grouping variable. A multilevel model was necessary to control for the nested structure of the data [30]. Furthermore, it allows the possibility of using country-level indicators to examine the relation between macro-level phenomenon and individual-level outcomes. We use intraclass correlation (ICC) to determine the explained variance of group-level variables, presented as a percentage of explained between-country variance in the models where country-level variables were included. The formula is (1-(ICC_m_/ICC_b_))*100, where ICC_m_ indicates the ICC of the model where a country-level variable has been included, and ICC_b_, the ICC of the baseline comparison model without the group-level variable.

With only 21 s-level units, the standard errors may be downwardly biased [31]. We therefore included only 1 s-level variable at a time to reduce the impact of biased results in the analysis. The analyses were post-stratification weighed.

## Results

Table 1 shows post stratification-weighted prevalences by the independent variables and the sizes of each subpopulation. Females, more than men, were found to report greater use of CAM. Prevalences were also found to be the highest among the age group 45–64, among those with higher education, and among those living comfortably on their income. However, for consumable CAM use we found the opposite relation with income. People in paid work were found to report greater CAM use than the unemployed and retired, while the permanently sick or disabled were found to report CAM use more than all other main activity groups. We found a clear pattern with regards to health care use, where having discussed own health with health care personnel, is related to all CAM categories. Similarly, people who reported having an unmet medical need, reported greater CAM use than those who did not. Those who reported being unsatisfied with health services in their country reported only marginally more CAM use than those of an average or higher opinion of health services, except for physical CAM use, where the relation was opposite. People who reported being in poor health also reported greater overall and consumable CAM use than people who reported being in good health, but we found no relation between health and physical CAM use. People who had reported having a longstanding health problem reported CAM use to a greater degree than people who did not.

**Table 1:** Distribution of sample, and prevalence by independent variables and the sizes of each subpopulation. Post-stratification weighted

Measure	Study population	Overall	Physical	Consumable
		CAM use	CAM use	CAM use
Total	33, 371	17.9%	10.9%	9.3%
Gender				
Male	47.3%	13.9%	9.0%	6.4%
Female	52.7%	21.5%	12.6%	11.9%
Age group				
25–44 years	37.6%	18.5%	11.6%	9.6%
45–64 years	38.4%	18.9%	11.9%	9.5%
65+ years	24.0%	15.3%	8.3%	8.4%
Educational level				
Primary education	32.6%	12.2%	6.8%	6.6%
Secondary education	49.3%	19.6%	12.1%	10.1%
Tertiary education	18.2%	23.2%	15.1%	11.8%
Financial strain				
Living comfortably	29.8%	20.7%	15.2%	8.2%
Coping	47.2%	17.2%	10.0%	9.6%
Difficult & very difficult	23.0%	15.5%	7.5%	9.9%
Main activity				
Paid work	57.2%	19.1%	12.3%	9.4%
Unemployed/housework	14.3%	16.7%	9.3%	10.1%
Retired	25.1%	15.1%	8.3%	8.3%
Permanently sick/disabled	3.4%	21.4%	14.9%	10.6%
Health care utilization				
No doctor visits	17.9%	8.8%	4.9%	4.6%
Only GP	39.9%	15.7%	9.2%	8.0%
MS/MS & GP	42.1%	23.7%	15.2%	12.5%
Unmet medical need				
No unmet need	87.5%	16.5%	10.2%	8.5%
Unmet need	12.5%	27.3%	16.3%	14.7%
Opinion of health services				
Average or higher opinion	68.8%	17.6%	11.1%	8.8%
Low opinion	31.2%	18.4%	10.6%	10.3%
Self-reported health				
Good health	64.6%	17.1%	11.0%	8.3%
Poor health	35.4%	19.3%	10.9%	11.0%
Longstanding health problem				
No longstanding health problem	71.7%	16.2%	9.8%	8.4%
Longstanding health problem	28.3%	22.1%	13.7%	11.5%

Table 2 presents four multilevel logistic models with individual-level variables. We found largely the same pattern as in the prevalence table. Females had used CAM to a greater degree than men after controlling for the other independent variables (OR = 1.62, 95% CI = 1.49–1.75). Age was not significantly related to CAM use. Higher education was related to greater use of all CAM categories (OR = 2.00, 95% CI = 1.70–2.34). Less financial strain was only statistically significant for physical and overall CAM use (physical OR = 1.46, 95% CI = 1.25–1.70). The same pattern was found with regards to employment, where the employed had used significantly more physical and overall CAM than the unemployed, but not consumable CAM (physical OR = 1.32, 95% CI = 1.10–1.58). Doctor visits were the strongest predictor for all types of CAM use. People who had visited a specialist were more likely to have used any CAM treatment than people who had not seen a doctor in the past year (OR = 2.87, 95% CI = 2.37–3.47). Unmet need was positively related to all types of CAM use (OR = 1.57, 95% CI = 1.40–1.76). Dissatisfaction with health services was positively related to all categories of CAM use (OR = 1.26, 95% CI = 1.17–1.36). SRH did not show a significant relation to any CAM use. Having a longstanding health problem was related to a higher use of all types of CAM use (OR = 1.44, 95% CI = 1.26–1.54). Lastly, the ICC indicates that the between-country variance were greater for the subgroups than for the overall measure (Overall ICC = 0.076, Physical ICC = 0.160, Consumable ICC = 0.158).

**Table 2:** Logistic multilevel models of CAM use with individual-level variables. Post stratification weighted

	Overall			Physical			Consumable		
	CAM use			CAM use			CAM use		
	O.R.	95% CI		O.R.	95% CI		O.R.	95% CI	
Female	1.62	1.49	1.75	1.44	1.28	1.62	1.80	1.61	2.01
Age group (ref. 25–44 years)									
45–64 years	1.01 (−)	.94	1.08	.97 (−)	.87	1.08	1.01 (−)	.93	1.11
65+ years	.94 (−)	.74	1.20	.81 (−)	.62	1.07	1.04 (−)	.79	1.36
Educational level (ref. Primary Education)									
Secondary education	1.58	1.39	1.79	1.68	1.46	1.93	1.48	1.24	1.77
Tertiary education	2.00	1.70	2.34	2.03	1.66	2.49	2.07	1.66	2.58
Financial strain (ref. Difficult/very difficult on present income)									
Coping	1.13 (−)	.98	1.31	1.24	1.07	1.45	1.06 (−)	.90	1.24
Living comfortably	1.31	1.15	1.49	1.46	1.25	1.70	1.10 (−)	.95	1.29
Main activity (ref. Unemployed/Housework)									
Paid work	1.15	1.03	1.29	1.32	1.10	1.58	.92 (−)	.81	1.06
Retired	.83 (−)	.69	1.00	.93 (−)	.75	1.15	.71	.58	.87
Permanently sick or disabled	1.03 (−)	.86	1.24	1.21 (−)	.97	1.50	.97 (−)	.75	1.26
Health care use (ref. No doctor visits)									
Only GP	1.84	1.62	2.10	1.76	1.53	2.02	1.87	1.59	2.20
MS or MS and GP	2.87	2.37	3.47	2.95	2.40	3.63	2.75	2.14	3.53
Unmet need	1.57	1.40	1.76	1.45	1.27	1.65	1.51	1.27	1.78
Dissatisfied with health services	1.26	1.17	1.36	1.33	1.22	1.45	1.21	1.08	1.35
Poor health	.97 (−)	.88	1.07	.97 (−)	.89	1.06	.98 (−)	.81	1.17
Longstanding health problem	1.44	1.26	1.65	1.54	1.32	1.79	1.29	1.11	1.51
Constant term	.03	.02	.04	.01	.01	.02	.02	.01	.03
ICC	.076			.160			.158		

(−) = Not significant on the .05 level

We performed two sensitivity analyses not presented in the tables, because the results were not in agreement with prior research. These results are available upon author request. First, we ran the models without controlling for health care utilization, unmet need, and longstanding health problems. Here, SRH was significantly positively related to physical treatments while controlling for health care utilization and unmet need, but not longstanding health problems. SRH was significantly positively related to consumable treatments while controlling for unmet need, but not health care utilization or longstanding illness. The second sensitivity analysis was to add an interaction between age groups and gender. The analysis showed that females aged 45–64 used the most physical CAM, while the men used less with higher reported age.

Table 3 shows the association between country-level variables and overall, physical, and consumable CAM use. Each row represents a new model with the indicated country-level variable included. Health expenditures per capita was positively related to overall and physical CAM use. Moreover, it was the best predictor for physical CAM use on the country level, with an explained between-country variance of 69%. Out-of-pocket payments were only significantly related to physical CAM use with a negative effect. Physician density was positively related to overall CAM use, but not significantly related to any of the sub-groups. Gatekeeping was negatively related to consumable CAM use, but not any of the other types of CAM use, making it the only significant predictor of between-country variance in consumable CAM use.

**Table 3:** Logistic multilevel models of CAM use with country-level variables. Post stratification weighted

	Overall		Physical		Consumable	
	CAM use		CAM use		CAM use	
Model with variable	Relation	Expl. Var.	Relation	Expl. Var.	Relation	Expl. Var.
Health exp. tot/capita	+	22%	+	69%		7%
OOP total		3%	–	23%		2%
Physicians density	+	18%		5%		4%
Gatekeeping		0%		0%		2%
Empty cell = Country-level variable not significant on the .05 level						

*Exp. Var*. Explained variance on the country level

## Discussion

This study aimed to examine the determinants of CAM use at the individual and country-level.

At the individual level, results indicate that females reported more overall CAM use than men, and that socioeconomic position (education, employment, and financial strain), in addition to longstanding illness, health care utilization, unmet medical needs and a negative opinion of the state of the health services were positively related to CAM use. These results were mostly replicated in the subgroup analysis of CAM treatments, with the exception that financial strain and employment were found to be significant predictors of physical but not consumable CAM use.

Prior studies have found greater CAM use among females [10, 32, 33]. Females have been found to report higher rates of unmet need [18], more health care utilization [34] and poorer health [35]. These were all factors predicting CAM use in our study. However, females still significantly used more CAM while controlling for these factors. This might indicate differences in values and personality traits such as risk seeking behavior, between men and women [36]. And in contrast to some previous work we did not find a relationship between CAM use and older age [8, 9], nor SRH [37]. Our sensitivity analysis revealed that middle-aged women reported the most physical CAM use, while men’s use decreased with older age. Therefore, the reason for age not showing a significant relation with CAM use might be that the male and female respondents, and the people who reported physical and those who reported consumable CAM use, pull the result in opposite directions, making the overall estimates not statistically significant. As for SRH, our sensitivity analysis showed that poor health was related to greater CAM use, before controlling for longstanding health problems, unmet need, and visits to health care practitioners, implying that SRH is an underlying factor for predicting CAM use. This result is in line with findings from Kemppainen et al. [10] who also find poor health to be a predictor for greater CAM use.

The results in terms of education and financial strain suggest the importance of individual resources in explaining CAM use. Prior research found education, employment and income to be related to CAM use [6, 8–10], and our results largely support this with one nuance: financial strain was not related to consumable CAM use in our data material. This finding might indicate that while physical CAM treatments generally involves paying and seeing a trained practitioner, the consumable treatments do not necessarily. People with more resources are better equipped to pay for more expensive CAM treatments, thus creating a social gradient in physical CAM use. It has also been suggested that people of a higher socioeconomic position may want to choose and control their approach towards health-related issues [5]. These results also support Astin’s [8] notion of educated people reading about possible treatments for their illness, challenging the doctor’s authority, and wanting to be in control of their own lives. However, it has been pointed out that even though users of alternative medicine may be better educated on average, it does not necessarily follow that they are better informed about the efficacy of alternative forms of treatment [5]. The physical CAM treatments are in part characterized by paying and seeing a trained practitioner for every treatment. For consumable treatments, this may not always be the case and might explain why they were not found to be related to financial strain and employment.

In terms of the health care explanatory variables, our results align with previous work which found that a quarter of the people who had used some form of CAM in the past year were referred by a conventional health care practitioner [20]. Some studies have shown that even though users of alternative health care almost make twice as many visits to conventional medical providers as non-users make, they are still reporting much higher levels of unmet need for health care [38]. This supports the conclusion of Druss and Rosenheck [39] that use of alternative treatments appears as a complement and not as an alternative to conventional health care.

At the country level, CAM use was best predicted by health expenditures. In the overall model, health expenditures explained around 22% of the between-country variation, while for physical CAM, the model explained around 69%. Results for consumable CAM treatments were non-significant. The high explained variance in physical treatments, and non-significant result for consumable treatments might be understood by looking at what specific treatments are reimbursed through health insurances and integrated into the established health care system. Neither countries in the high nor low end of health expenditure have widely integrated homeopathy, or herbal treatment in the health care system or reimbursement through health insurance [14]. The treatments that have been integrated or reimbursed in the high health expenditure countries are acupuncture and chiropractics, both physical treatments. Higher integration is therefore closely related to health expenditures, and health expenditures explains 69% of the variation in physical CAM use. Health expenditures is best predicted by GDP [40], suggesting the underlying factor is the economy of the countries, making a more diverse selection of treatments, including CAM, available for the public.

To our knowledge this work is the first to examine country-level determinants of CAM use. The finding that healthcare expenditure explains much of the intercountry variance of physical CAM use has in common with the individual-level results that resources seem to be an important predictor of CAM use. The physical treatments cost more on average than the consumable treatments. This might play a role in explaining why the less financially strained used more physical, but not consumable CAM than those who were more financially strained. On the country level, having more resources gives the same outcome as on the individual level. The exact mechanism is still unclear, but one could hypothesize that the process of integrating CAM into the established health care systems requires resources, and that the physical treatments costs more to integrate due to more equipment and education required to make that happen. Furthermore, treatments which require trained practitioners might be easier to incorporate into the health care system, as it makes accountability possible because the training is formalized. Richer countries may therefore be better equipped to make the integration happen.

### Limitations

This study should be interpreted in the light of some limitations. Although the ESS maintains a high standard of data collection, the survey is still prone to differences in response rates, and cross-cultural quality of questions [11]. The ESS uses cross-sectional data, and therefore it is difficult to draw conclusions with regards to causal relationships. For example, dissatisfaction with health care may influence people to use CAM, but the causal relationship might also go the other direction. There are also some methodological limitations related to our work; e.g. the data used in the analyses only measure whether respondents have used CAM or not in the past year, and does not provide information on the frequency of care. A person using CAM weekly would preferably be considered differently than a person using such treatments once a year.

## Conclusion

At the individual level, we found CAM use to be associated with a range of socioeconomic, demographic and health indicators. At the country level, we found that countries’ health expenditures were positively related to the prevalence of overall and physical CAM treatments. Therefore, a common predictor for CAM use, both at the individual and country-level, is greater resources. At the individual level greater resources may influence CAM use through an out-of-pocket payment for the wished treatment, making the less resourceful less equipped to seek it. At the country level, greater resources may be related to how well CAM is integrated into conventional health care systems. Based on these conclusions, a hypothesis for future research would be that countries with less CAM integration in the health care system, would have a steeper social gradient with regards to income than countries with more reimbursements through health insurances and more CAM integration.

## Supplementary information

**Additional file 1: Appendix**. Participating countries’ average CAM use and second-level variables.

## Data Availability

The ESS7–2014 Edition 2.2 was released on 1 December 2018. The data file can be downloaded here: https://www.europeansocialsurvey.org/data/download.html?r=7. Please note that users are obliged to read the ESS conditions of use: https://www.europeansocialsurvey.org/data/conditions_of_use.html.

## Abbreviations

CAM: Complementary and alternative medicine
CI: Confidence interval
ESS: European Social Survey
ICC: Intraclass correlation
ISCED: International Standard Classification of Education
NCCAM: National Center for Complementary and Alternative Medicine
OR: Odds ratio
OECD: The Organisation for Economic Co-operation and Development
*SRH*: *Self-reported health*
