# Supplementary material for: The use of complementary and alternative medicine (CAM) in Europe
Source: BMC Complement Med Ther. 2020 Apr 6;20:108. doi: 10.1186/s12906-020-02903-w (PMC7137515; doi:10.1186/s12906-020-02903-w)
Supplement: Supplementary file 1 — Additional file 1: Appendix. Participating countries’ average CAM use and second-level variables. [file 12906_2020_2903_MOESM1_ESM.docx]

Appendix: Participating countries’ average CAM use and second-level variables.

|  | Overall | Physical | Consumable | Health exp. tot. | Out-of-pocket | Physician |  |
| --- | --- | --- | --- | --- | --- | --- | --- |
| Country | CAM use | CAM use | CAM use | per capita | payments | density | Gatekeeping |
| Poland | .06 | .02 | .05 | 910.28 | 23.46 | 2.22 | 1 |
| Portugal | .07 | .04 | .04 | 2096.82 | 26.84 | 4.10 | 1 |
| Hungary | .07 | .03 | .06 | 1036.62 | 26.59 | 3.08 | 1 |
| Netherlands | .11 | .08 | .05 | 5693.86 | 5.22 | 2.86 | 1 |
| Spain | .12 | .07 | .07 | 2658.27 | 24.00 | 4.95 | 1 |
| Israel | .12 | .11 | .04 | 2910.29 | 26.98 | 3.34 | 0 |
| Ireland | .13 | .11 | .03 | 4239.15 | 17.66 | 2.67 | 1 |
| Slovenia | .14 | .06 | .11 | 2160.75 | 12.07 | 2.52 | 1 |
| Finland | .14 | .12 | .04 | 4612.29 | 18.23 | 2.91 | 1 |
| Czech Republic | .15 | .04 | .13 | 1378.52 | 14.33 | 3.62 | 0 |
| Great Britain | .15 | .11 | .06 | 3934.82 | 9.73 | 2.81 | 1 |
| Belgium | .19 | .14 | .09 | 4884.07 | 17.81 | 4.89 | 0 |
| Sweden | .20 | .18 | .02 | 6807.72 | 14.06 | 3.93 | 1 |
| Norway | .21 | .20 | .02 | 9522.22 | 13.61 | 4.28 | 1 |
| Austria | .21 | .10 | .14 | 5580.49 | 16.15 | 4.83 | 0 |
| Germany | .23 | .15 | .12 | 5410.64 | 13.20 | 3.89 | 1 |
| Estonia | .24 | .07 | .20 | 1248.28 | 20.72 | 3.24 | 1 |
| Denmark | .25 | .23 | .03 | 6463.24 | 13.36 | 3.49 | 1 |
| Switzerland | .29 | .20 | .14 | 9673.52 | 26.80 | 4.05 | 1 |
| Lithuania | .30 | .02 | .29 | 1063.42 | 31.27 | 4.12 | 1 |
| France | .30 | .21 | .16 | 4958.99 | 6.34 | 3.19 | 1 |
